# Effect of single-incision laparoscopic distal gastrectomy guided by ERAS and the influence on immune function

**DOI:** 10.1186/s12957-021-02422-z

**Published:** 2021-10-20

**Authors:** Junfeng Zhou, Sheng Lin, Sida Sun, Chengying Zheng, Jiaxing Wang, Qingliang He

**Affiliations:** 1 Department of Gastrointestinal Surgery, The First Affiliated Hospital of Fujian Medical University, No.20 Chazhong Road, Fuzhou, 350005 Fujian PR China; 2 Department of Pediatric Surgery, Fujian Children’s Hospital, Fujian 350005 Fuzhou, PR China

**Keywords:** Single-incision laparoscopic distal gastrectomy, Multiport laparoscopic distal gastrectomy, Enhanced recovery after surgery, Gastric cancer, Immune function

## Abstract

**Background:**

To evaluate the immune function of gastric cancer patients after single-incision laparoscopic distal gastrectomy (SIDG) or multiport laparoscopic distal gastrectomy (MLDG) guided by enhanced recovery after surgery (ERAS).

**Methods:**

A retrospective cohort study was performed on 120 patients who underwent laparoscopic distal gastrectomy for gastric cancer. The patients were divided into two groups according to operation method: group A (MLDG) and group B (SIDG), both guided by ERAS concept. The indicators reflecting immune function and inflammation, such as CD3^+^, CD4^+^, CD8^+^ and NK cell count, CD4^+^/CD8^+^ cell ratios, IgA, IgM and IgG levels, C-reactive protein (CRP), total lymphocyte count (TLC) and neutrophil-to-lymphocyte ratio (NLR) were tested 3 days and 7 days after surgery.

**Results:**

The skin incision length of patients in group B was significantly shorter than that in group A, but the operation time was significantly longer in group B than that in group A (*P* < 0.05). There were no significant differences in preoperative CD3+, CD4+, CD8+, natural killer (NK) cells, CD4+/CD8+, IgA, IgM and IgG levels between two groups (*P* < 0.05). Three days after surgery, the immune function indices were decreased in both groups, but with no significant difference between two groups (*P* > 0.05). On the 7th day after surgery, the immune indexes of both groups recovered somewhat, approaching the preoperative level (*P* > 0.05). Inflammation indexes increased 3 days after surgery and decreased 7 days after surgery in both groups, among them the CRP level in group A was higher than that in group B (*P* < 0.05). The 3-year survival rate were 96.7% in group A and 91.7% in group B, respectively, with no statistically significant difference.

**Conclusion:**

Compared with MLDG guided by ERAS, SIDG under the guidance of the ERAS concept has better cosmetic effect and similar effect on immune function of gastric cancer patients.

## Introduction

Gastric cancer is still a high incidence tumor and a major cause of cancer death globally [1]. With the progress of economy and technology, the detection rate of early gastric cancer in China has been increasing year by year, from less than 10% to nearly 20% [2]. Laparoscopic distal gastrectomy (LDG) has become one of the standard surgical procedures for treatment of early distal gastric cancer [3]. Some studies reported the implementation of enhanced recovery after surgery (ERAS) can reduce the degree of immunosuppression and improve the long-term prognosis of cancer patients [4, 5]. The single-incision laparoscopic surgery (SILS) has certain advantages in reducing trauma and shortening postoperative recovery time, which well fits the concept of ERAS [6, 7]. With the rise of SILS and the application of the concept of ERAS, a new door has been opened for minimally invasive treatment of gastric cancer patients [8, 9].

The occurrence, development and prognosis of tumors are closely related to the cellular immune status of human T lymphocytes. Natural killer (NK) cells play an extremely important role in anti-tumor immunity, CD3^+^ T cells can reflect the immune state of the body, CD4^+^ T cells are auxiliary and inductive T cells, and CD8^+^T cells are inhibitory T cells. The ratio of CD4^+^/CD8^+^T cells is an important indicator reflecting the immune regulatory function of the body [10]. Humoral immunity, on the other hand, activates the complement through the combination of antibodies and tumor antigens, leading to cell lysis and antibody-mediated conditioning to play an anti-tumor immune effect mechanism [11]. Furthermore, the total lymphocyte count (TLC), neutrophil to lymphocyte ratio (NLR) and C-reactive protein (CRP) are immune-related inflammatory indicators, which have been proved to be correlated with the prognosis of tumor patients [12, 13]. Ma et al. have reported that compared with open radical gastrectomy, LDG has less impact on the immune system and lower inflammatory response of gastric cancer patients [14].

One study has shown that there was no significant difference in terms of intraoperative bleeding, operative time, number of lymph nodes dissected and survival rate between SILS and traditional multiport laparoscopic surgery (MPLS) for gastric cancer patients [15]. While, SILS has the inherent advantages of minimally invasive and quick recovery, thus well reduce the stress of patients [15]. There are few reports on the effect of both methods on immune function of gastric cancer patients. Further, the 3-year survival rate for early gastric cancer has been reported > 90% [16]. Therefore, this study aims to further explore the effect of ERAS concept guided single-incision laparoscopic distal gastrectomy (SIDG) versus traditional multiport laparoscopic distal gastrectomy (MLDG) on immune function of gastric cancer patients, and further analyze the 3-year survival rate of patients.

## Materials and methods

### Patients

A retrospective cohort study was performed on 120 patients who underwent LDG for gastric cancer at our hospital from January 2014 to December 2016. The patients were divided into two groups according to operation method: group A was performed traditional MLDG guided by ERAS concept; group B was performed SIDG guided by ERAS concept. The study was approved by the local research ethics committee of our hospital (the approval number: MRCTA, ECFAH of FMU [2019] 162) followed international and national regulations in accordance with the Declaration of Helsinki. Written informed consent was obtained from all patients allowing us to store their data in our hospital database and use it for clinical research.

Inclusion criteria were: (1) Tumor clinical stage was I A or I B according to the seventh edition of American Joint Committee on Cancer (AJCC) [17]; (2) Conventional LDG and SIDG were performed along with D1 or D1+ lymph node dissection; (3) All the operations were performed by the same surgeon. Exclusion criteria were: (1) Patients had a history of gastric surgery; (2) Patients with conversion to open surgery; (3) Patients with BMI > 30 kg/m^2^ or BMI < 15 kg/m^2^; (4) Patients were at ASA IV or V stage, according to the American Society of Anesthesiologists classification (ASA) [17].

### Surgical treatment

Perioperative protocol of ERAS mainly referred to the ERAS® Society [18, 19]. D1 and D1+ lymph node dissection were performed according to the Japanese Gastric Cancer Treatment Guidelines. Conventional MPLS was performed according to Laparoscopic Operation Guidelines for Gastric Cancer [20]. Laparoendoscopic single-site surgery (LESS) inserted a single-port converter (Meiwai Company, Shanghai, China) through a transumbilical incision. The detail technical tips for SIDG was based on our previous published study [19]. The discharge criteria was: had no pain with oral analgesics, could take the semi liquid food, required no intravenous rehydration, walk freely, and acceptance of discharge by the patient. Hospital readmission for any postoperative complication occurring within 30 days after discharge was recorded.

### Observation index

The surgical situation, clinical data, changes in immune indexes and postoperative survival time of the two groups were collected and compared. Preoperative risk assessment of patients was based on the ASA classification [21]. Intraoperative indicators included operation time, intraoperative blood loss, the length of the incision and number of resected lymph nodes. The operation time was calculated from making an incision to stitch incision. Postoperative indicators included ambulation time, time to recovery of bowel function, time to semi-liquid diet, postoperative hospital stay and survival time. Fasting venous blood samples were collected from all cases. The indices of immune function (CD3^+^, CD4^+^, CD8^+^ and NK cell count, CD4^+^/CD8^+^ cell ratios, IgA, IgM and IgG levels) and inflammation (CRP, TLC, NLR) were tested in the two groups preoperatively, 3 days and 7 days after surgery.

### Statistical analysis

Statistical analyses were performed by using SPSS version 21.0 (SPSS Inc., Chicago, Illinois). The normal distribution of the measurement data was expressed by means ± standard deviation (SD). Continuous outcome variables were analyzed using the t test or Mann-Whitney U test. Discrete variables were analyzed with the Chi-square test or Fisher exact test. Rank sum test was used for the analysis of rank data at appropriate. Kaplan-Meier method was used to establish survival curves, and log-rank test was used to analyze the survival curves. If the *P* value was less than 0.05, it was considered as statistically different.

## Results

### Characteristics of patients

Patients were randomly divided into group A (*n* = 60) and group B (*n* = 60). Differences between the two groups in terms of age, gender, body mass index (BMI), lymph node resection, tumor differentiation TNM pathological stage was of no statistical significance (all *P* > 0.05, Table 1).

**Table 1:** Clinical characteristics of patients

Characteristics	Group A (*n*=60)	Group B (*n*=60)	*P*-value
Age (years)	62.3±7.5	62.8±7.3	0.555
Gender (n)			0.583
Male	34	30	
Female	26	30	
BMI (kg/m^**2**^)	21.6±2.5	21.9±2.6	0.750
ASA grade (n)			0.518
I	44	48	
II	16	12	
III	0	0	
Lymph node resection (n)			0.825
D1	12	14	
D1+	48	46	
TNM stage (n)			0.777
IA	54	52	
IB	6	8	
Differentiation (n)			0.919
High differentiated	12	12	
Moderately differentiated	26	28	
Poorly differentiated	22	20	

Data presented as mean ± SD. *ASA* American Society of Anesthesiologists, *BMI* Body Mass Index, *TNM* Tumor, Node, Metastasis

### Operation index

The skin incision length of patients in group B was significantly shorter than that in group A, but the operation time was significantly longer in group B than that in group A (all *P* < 0.05). There was no significant difference in terms of the amount of intraoperative blood loss and the number of lymph nodes dissected between the two groups (all *P* > 0.05). Meanwhile, there were no significant differences in terms of postoperative ambulation time, time to recovery of bowel function, time to semi-liquid diet, postoperative hospital stay between the two groups (all *P* > 0.05), as shown in Table 2.

**Table 2:** Comparison of intraoperative and postoperative indexes between the two groups

Indexes		Group A (*n*=60)	Group B (*n*=60)	*P*-value
Operation time (min)		185.5±15.5	215.3±23.4	<0.001
Blood loss (ml)		129.1±17.3	138.9±31.1	0.057
Skin incision (cm)		11.3±1.1	5.3±1.0	<0.001
Number of resected lymph nodes		19.4±2.5	18.6±1.9	0.071
Ambulation time (d)		2.3±0.6	2.1±0.7	0.069
Time to semi-liquid diet (d)		3.2±0.6	3.2±0.7	0.784
Time to recovery of bowel function (d)		3.1±0.7	3.0±0.6	0.211
Postoperative hospital stay (d)		5.5±1.1	5.9±1.4	0.097

### Immune cell and inflammation testing results

There were no significant differences in terms of preoperative CD3^+^, CD4^+^, CD8^+^, NK cell count, CD4^+^/CD8^+^ cell ratios, CRP, TLC, NLR, IgA, IgM and IgG levels between two groups (*P* < 0.05). Three days after surgery, the immune indexes in both groups decreased, but the difference between the two groups was not statistically significant (*P* > 0.05). On the 7th day after surgery, the immune indexes of both groups recovered somewhat, approaching the preoperative level, and there was no statistical significance between the two groups (*P* > 0.05). Further, the inflammation indexes increased 3 days after surgery and decreased 7 days after surgery in both groups, among them the CRP level in group A was higher than that in group B (*P* < 0.05) (Table 3).

**Table 3:** Comparison of immune cell testing results in the two groups

Index	Group A	Group B	*P*-value
CD4^+^ (%)			
Preoperative	40.6±3.5	41.8±3.3	0.052
Three days after surgery	31.8±6.7	31.1±5.5	0.533
Seven days after surgery	35.8±5.7	37.5±4.1	0.068
CD8^+^ (%)			
Preoperative	31.4±2.6	31.1±2.7	0.547
Three days after surgery	19.3±4.6	20.0±4.4	0.442
Seven days after surgery	27.0±4.1	27.8±3.4	0.266
CD4^+^/CD8^+^			
Preoperative	1.3±0.1	1.4±0.2	0.063
Three days after surgery	1.7±0.6	1.6±0.4	0.174
Seven days after surgery	1.4±0.3	1.4±0.2	0.761
CD3^+^ (%)			
Preoperative	61.9±4.2	60.5±4.2	0.065
Three days after surgery	47.6±3.4	46.6±3.5	0.108
Seven days after surgery	60.0±3.3	59.4±3.1	0.252
NK cell (10^9/L)			
Preoperative	0.282±0.02	0.284±0.02	0.777
Three days after surgery	0.242±0.03	0.240±0.02	0.688
Seven days after surgery	0.263±0.03	0.260±0.03	0.437
IgA (IU/ml)			
Preoperative	153.3±29.8	155.1±24.9	0.715
Three days after surgery	124.2±21.9	122.9±20.3	0.730
Seven days after surgery	140.0±26.1	136.5±22.7	0.440
IgM (IU/ml)			
Preoperative	175.3±26.4	175.3±28.0	0.995
Three days after surgery	143.3±22.5	146.0±24.3	0.531
Seven days after surgery	158.3±23.2	160.8±25.7	0.572
IgG (IU/ml)			
Preoperative	137.2±22.2	135.5±20.5	0.667
Three days after surgery	110.0±19.6	111.6±17.4	0.627
Seven days after surgery	122.3±21.4	123.9±18.8	0.674
CRP (mg/L)			
Preoperative	3.7±1.1	4.0±1.1	0.173
Three days after surgery	59.2±13.0	53.3±11.2	<0.01
Seven days after surgery	32.4±9.0	28.4±9.0	0.015
TLC (10^9/L)			
Preoperative	1.8±0.5	1.7±0.3	0.099
Three days after surgery	2.5±0.5	2.5±0.5	0.778
Seven days after surgery	2.7±0.5	2.7±0.4	0.665
NLR			
Preoperative	1.9±0.2	2.0±0.2	0.056
Three days after surgery	3.2±0.8	3.0±0.7	0.249
Seven days after surgery	2.0±0.4	2.0±0.4	0.525

NK Natural killer, CRP C-reactive protein, TLC Total lymphocyte count, *NLR* Neutrophil-to-lymphocyte ratio

### Three-year survival rate after surgery

**Fig. 1:**
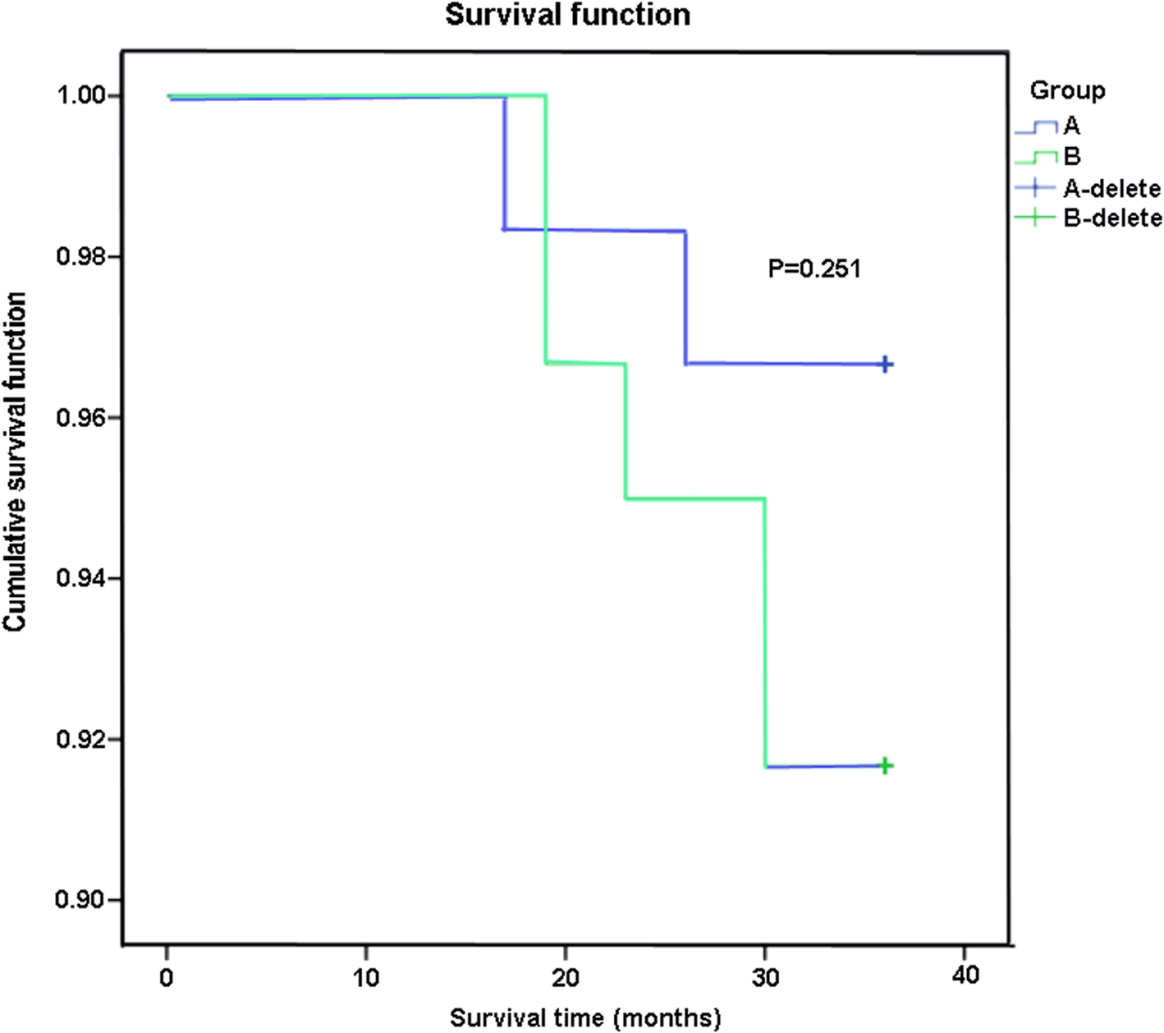
The 3-year survival rate of two groups

The 3-year survival rate after surgery was shown in Fig. 1. The 3-year survival rate of patients were 96.7% in group A and 91.7% in group B, respectively. And, there was no statistically significant difference in 3-year survival rate between the two groups (*P* > 0.05).

## Discussion

With people’s attention on self-health, the detection rate of early gastric cancer has been increasing year by year [22]. The efficacy of laparoscopic surgery in early gastric cancer has been recognized, and it has the advantages of small incision, less bleeding, quick recovery, and is safe and reliable in the radical effect. On this basis, the technique of single-port laparoscopy arises at the historic moment, with better postoperative cosmetic effect and lower postoperative pain [23]. In 2011, Omori T et al. [24] first reported radical transumbilical single-port laparoscopic resection for early gastric cancer, confirming it was safe and feasible, and has good cosmetic effect. Although the perioperative management of gastric cancer patients is still controversial, ERAS can significantly shorten the length of postoperative hospital stay, reduce complications and improve organ function [25], which has been supported by more and more evidence-based medicine. For example, the European Association of Accelerated Rehabilitation Surgery formulated the Guidelines for Accelerated Rehabilitation Surgery in Gastrectomy in 2014 [18]. In 2016, the Chinese Accelerated Surgery Expert Group published the Chinese expert consensus on the perioperative management of accelerated rehabilitation surgery [26].

The immune response of an organism to tumor is primarily mediated by T cells. T-cell subsets such as CD3+, CD4+, CD8+ and CD4+/CD8+ can directly reflect the postoperative immune functions of cancer patients. Kehlet et al. [27] has reported laparoscopic surgery has less suppression to the immune function of patients. There were studies also have shown that compared with open surgery, laparoscopic surgery has better protection for the immune function of patients, mainly due to the surgical methods [28–30]. There are few studies on whether SIDG has further advantages over MLDG in maintaining postoperative immune homeostasis and reducing immune suppression in gastric cancer patients. In this study, both the cellular immunity and humoral immunity indexes of patients in the two groups decreased after surgery, reflecting the impact of surgical trauma on the immune homeostasis of patients. On the 7th day after surgery, all the immune indexes had rebounded, and there was no statistical significance between the two groups, indicating both have certain protection for immune function. The result indicated compared with MLDG, SIDG was mainly embodied in the smaller incision length, better cosmetic effect.

The relapse and metastasis of malignant tumor after surgery are closely related to immune status. The relationship between inflammatory indexes such as TLC, NLR and CRP and tumor short-term and long-term prognosis has attracted more and more attention. Studies have shown that the increase of postoperative inflammatory indicators was negatively correlated with tumor prognosis, the higher level of NLR led to the greater possibility of lymph node metastasis [31, 32]. Stress response is closely related to immune function, prolonged stress state or excessive stress leads to immunosuppression. CRP is an important acute inflammatory mediator, and commonly used in studying stress response. In this study, the inflammatory indicators (TLC, NLR and CRP) of the two groups correspondingly increased 3 days after surgery and decreased 7 days after surgery, reflecting the trauma and stress-related symptoms in operation. However, there was no significant difference in the postoperative levels of TLC and NLR between the two groups, while the postoperative CRP level of the SIDG group was lower than that of MLDG group. One possible reason is that the SIDG has the advantage of minimally invasive, smaller wound and less pain, more comfortable postoperative experience, which could effectively reduce the inflammatory stress response of patients.

Wichmann et al. have shown that the concept of ERAS can better protect cell-mediated immune function, because strong stress response can inhibit the cellular immune function of the body, and the degree of inhibition is positively correlated with the size of trauma [33]. A series of measures of ERAS effectively alleviated the stress of patients, such as shortening the time of fasting water and early postoperative eating effectively alleviated the perioperative “hunger and thirst” state of patients, good pain management, and early ground movement, which may effectively protect the homeostasis of their internal environment, thus reduce the suppression of immune function of patients caused by surgical trauma. This study suggests that the ERAS concept can significantly reduce patients’ traumatic stress and play an effective protective role in patients’ immune function.

## Conclusion

In general, compared with traditional MLDG guided by ERAS, SIDG under the guidance of the ERAS concept has better cosmetic effect and similar effect on immune function of gastric cancer patients. The prospective randomized controlled trials with a larger sample size are still needed to further determine the many advantages of SIDG under the guidance of the ERAS concept in early gastric cancer.

## Data Availability

All data generated or analyzed during this case report were included in this published article.

## Abbreviations

SIDG: Single-incision laparoscopic distal gastrectomy
MLDG: Multiport laparoscopic distal gastrectomy
ERAS: Enhanced recovery after surgery
LDG: Laparoscopic distal gastrectomy
NK: Natural killer
SILS: Single-incision laparoscopic surgery
MPLS: Multiport laparoscopic surgery
CRP: C-reactive protein
TLC: Total lymphocyte count
NLR: Neutrophil-to-lymphocyte ratio
AJCC: American Joint Committee on Cancer
ASA: American Society of Anesthesiologists
LESS: Laparoendoscopic single-site surgery
BMI: Body mass index
